# Laryngeal Diphtheria, with Special Reference to Intubation

**Published:** 1907-03

**Authors:** T. J. Dodson

**Affiliations:** Mangum, Okla


### TEXAS MEDICAL JOURNAL.

Established July, 1885.

F. E. DANIEL, M. D., - - - - Editor, Publisher and Proprietor.

PUBLISHED MONTHLY.—SUBSCRIPTION $1.00 A YEAR.

VOL. XXII. AUSTIN, MARCH, 1907. No. 9.

The publisher is not responsible for the views of contributors.

For Texas Medical Journal.

### Laryngeal Diphtheria, With Special Reference to Intubation.

BY T. J. DODSON, M. D., MANGUM, OKLA.

Laryngeal diphtheria may be considered under the following names: Pseudo-membranous laryngitis, membranous croup, true diphtheria, true croup, and laryngeal stenosis. For nearly a half-century the identity of membranous croup and laryngeal diphtheria has been contended for by some and denied by others equally as good. Bacteriology has settled many questions along this line; statistics showing that 80 per cent of these cases are true diphtheria; i. e., it is due to the Klebs-Loeffler bacillus.

In cases of primary laryngeal diphtheria, there are wanting many of the characteristic features which distinguish diphtheria of the pharynx. There are two reasons for this: one is the rapid course of the disease, often producing death from local causes before the constitutional symptoms resulting from the absorption of toxin have developed. The second reason is, that the absorption of poison by the laryngeal mucous membrane is very slow and feeble as compared with that which takes place from the pharynx.

It is unnecessary for me to enter into the differential diagnosis between the different forms of laryngeal stenosis, for as has already been stated, 80 per cent of all cases show the Klebs-Loeffler bacillus; we will, therefore, consider them as laryngeal diphtheria. Neither will I consume your time by going over the long list of symptoms which characterize this disease, as it is the intention of this paper to deal with those cases only which have advanced to that stage where operative measures must be considered. I am sure that any one who has ever seen a case of this kind once, would hardly make a mistake in his diagnosis.

That there are cases, whether it be diphtheria or not, where nothing but operative measures promise relief, none will deny. Opinions will differ as to the time when operative interference is called for. One should never wait for general cyanosis, for often this does not occur until just before death; it is better to operate too early than too late.

After a fair trial has been made of other remedies, and if, in spite of all this, the dyspnea continues to increase steadily and the temperature begins to rise, operation should not be deferred longer. When this has been decided upon, the physician has his choice between intubation and tracheotomy. During the last ten years intubation has grown steadily in favor, and since the introduction of antitoxin, tracheotomy has been practically abandoned, it being resorted to only in rare cases, after intubation has failed to give relief.

A report of the American Pediatric Society says: "Before the use of antitoxin 27 per cent of intubation cases recovered, now 73 per cent recover; and an early use of antitoxin will lower this mortality still more. Sixty per cent of stenosis cases do not require operation if antitoxin is in time. My personal experience with intubation has been very satisfactory, indeed, a report of which I will give later. In combination with antitoxin I consider intubation one of the greatest blessings at the disposal of the physician. The operation of intubation and extubation is not in itself difficult, the latter being more difficult than the former. Only two assistants are necessary, neither of whom need necessarily be a physician, and proceed as follows: Remove all clothing from the child except the underclothing, and wrap the child securely in a sheet from shoulders down, secured by safety pins. Place the child upright, facing the operator, in the lap of one assistant, who sits upright in a common straight-backed chair; the arms of the patient should be firmly held below the elbows; the child's legs are clasped between the knees of the assistant. The second assistant stands behind the chair and when the gag is introduced, includes it within the firm grasp. The position of the child should be as though it hung from the top of its head. The operator should have the tube properly prepared, and armed with a small string; this is necessary in case the tube should become blocked or be turned loose in the esophagus, it can be immediately removed. The operator now stands in front of the patient and inserts the index finger of the left hand, passing it well back into the pharynx, then bringing it forward until the upper border of the cricoid cartilage is felt; directly in front of the cricoid cartilage may be felt the epiglottis and the opening into the larynx. The epiglottis is hooked up, and with the tongue brought forward and upward, the tube is passed along the palmar surface of the index finger and guided into the larynx, when the tube has reached the opening of the larynx; the handle of the operator should be well elevated in order to prevent the tube from passing over the larynx and going into the esophagus. The tube is then released from the introducer and gently pushed home by the finger.

Any carelessness in carrying out these details may result in failure to introduce the tube. When the tube is in the larynx and not blocked by detached membranes, a characteristic moist rattle will be heard as the air passes in and out in respiration. If a detached membrane has been forced down, the child will become more cyanotic, when the tube should be pulled out by the string and re-introduced when the detached membrane has been expelled by coughing. (In one case I introduced the tube the third time before it was allowed to remain.) When the paroxysm of coughing is over, which is usually excited by the tube in the larynx, and the dyspnea is relieved, and there is no evidence of detached membrane below the tube, the string and then the gag may be removed, but if there is still some evidence of detached membrane below the tube, it is better to leave the string fastened to the cheek by a piece of adhesive plaster. If the tube should be coughed up after having been in the larynx a day or two, a re-introduction is not necessary until urgent symptoms demand it. Feeding is generally not very difficult; some children swallow liquids easily, while others swallow semi-solids best.

Tubes may be removed after two, four or six days; when it is seen that a greenish muco-pus is coughed up through the tube it is time to remove it. This is rather more difficult than its introduction. The general arrangements of the patient and the assistants are the same as that of introduction. The left index finger is placed upon the head of the tube, which is steadied externally by the thumb of the same hand; the beak of the extractor is introduced into the opening of the tube, and it is gently withdrawn, very little force being required. If the dyspnea does not return in three or four hours the probabilities are that the tube will no longer be required. It is very exceptional that the patient has any difficulty in dispensing with the tube, as is so often the case in tracheotomy.

The advantages of intubation over that of tracheotomy, claimed by those who have had considerable experience in both operations, are as follows: (1) It is quicker, safer, simpler and adds no danger to the original disease; (2) there is no shock or hemorrhage; (3) no anesthetic is required nor trained assistants necessary; (4) no fresh wound is made which may prove an avenue of infection; (5) it gives an opportunity for a better expulsive, which is of great value in dislodging false membrane and mucus; (6) there is no objection on the part of the parents to be overcome; (7) the air is warmed and moistened, as it is normally, by passing over the nasal and buccal mucous membranes; (8) no skilled after-treatment is required; (9) in infancy, especially, all who have had considerable experience in both operations, admit the great superiority of intubation; (10) the tube can be dispensed with earlier and with much less difficulty; (11) if tracheotomy is required later on, the tube does not interfere with the operation.

Experience has proved that intubation does relieve the dyspnea in laryngeal stenosis promptly, effectually and certainly, and does not deprive the patient of any advantage that tracheotomy offers.

### REPORT OF CASES.

No. 1. Called to see child of Henry H., age 3½ years, on Saturday, October 6th, at 2 o'clock p. m. Diagnosis, laryngeal diphtheria with progressive stenosis; gave 2000 units antitoxin and in addition gave the usual course of calomel. Next morning at 9 a. m. no improvement; gave 3000 units antitoxin; at 11 o'clock intubation was done, the stenosis completely relieved. The child continued to rest well and sleep well. On the following Wdnesday night at 11 o'clock the tube was coughed up; the dyspnea did not return, and the tube was not re-introduced. The child made a rapid and complete recovery.

No. 2. A Greek child about 5 years old, had laryngeal stenosis for three days; was intubated November 8th. In this case the membrane was so thick and the mucus so excessive that the tube was withdrawn and re-introduced the third time before it was allowed to remain. This tube remained in the larynx six days. The child made a slow but successful recovery. In this case there was no antitoxin used.

No. 3. Child 4 years old; was seen with Dr. S. on November 28th. So urgent was this case that I received two hurried phone calls on the road, fearing the child would die before relief could be given. The tube was introduced and immediate relief followed.

Three thousand units of antitoxin was given at once and 3000 more the following day. This was the only time the child was seen by a physician until the fourth day, when the tube was removed, and the child made an uninterrupted recovery.

No. 4. Child 5 years old; was intubated December 18th. The tube remained in the larynx four days, when it was brought to town, a distance of twenty-five miles. The tube was removed, and the child made a rapid recovery.

No. 5. This child was about 4 years old, and evidently had a secondary attack, the first one being of the pharyngeal type, the second one coming on about ten days after the first, but before the child had made a complete recovery; it took no antitoxin the first time, but ten days later it was taken with laryngeal diphtheria with progressive stenosis. Intubation gave complete relief, but the tube was coughed out thirty-six hours after being introduced; however the troublesome stenosis did not return and the tube was allowed to remain out. The child had several doses of antitoxin, but made a very slow recovery.

No. 6. A boy 6 years old; gave a history of repeated attacks of spasmodic croup, but at this particular time was taken one night and the stenosis continued during the following day, and by the second night the stenosis seemed almost complete; in fact, the breathing was as bad, if not worse, than any case I have seen. Intubation was done and 3000 units of antitoxin given; the stenosis was completely relieved, and the child slept nicely for six hours, when, during a paroxysm of coughing, the tube was coughed up, and as the stenosis did not return, the tube was allowed to remain out. This, in my opinion, was a case of simple stenosis and not one of laryngeal diphtheria.

